# The association between test anxiety, learning strategies, and open-label placebo effects on academic test performance: a secondary analysis of a randomized controlled trial

**DOI:** 10.3389/fpsyg.2025.1529056

**Published:** 2025-10-09

**Authors:** Elisa Frisaldi, Julian Kleine-Borgmann, Helena Hartmann, Sven Benson, Ulrike Bingel, Katharina Schmidt

**Affiliations:** ^1^Department of Neurology, Center for Translational Neuro- and Behavioral Sciences, University Medicine Essen, University Duisburg-Essen, Essen, Germany; ^2^Institute for Medical Education, Center for Translational Neuro- and Behavioral Sciences, University Medicine Essen, University Duisburg-Essen, Essen, Germany

**Keywords:** open-label placebo (OLP), test anxiety, learning strategies, test performance, self-efficacy

## Abstract

**Introduction:**

The management of educational stressors and predictors of cognitive performance outcomes, such as test anxiety and learning strategies (LS), pose relevant challenges for students and educators. In a previously published single-blind randomized controlled trial (DRKS00013557), we reported on the impact of a 3-week open-label placebo (OLP) treatment compared to no intervention on results of a central examination and subjective well-being in healthy medical students. OLP treatment had a positive effect on students' subjective well-being, and test performance was better in those students in the OLP group with higher beliefs in benefits of medication. The present secondary analysis, conducted on a subgroup of the main study, aimed to explore whether further potential factors of exam performance influenced the impact of OLPs intake on cognitive outcomes.

**Methods:**

This secondary analysis investigated a subgroup of the main study's sample (*N* = 104) in which learning strategies were assessed. Here, we conducted an explorative analysis to investigate the effects of test anxiety, LS, and 3-week OLP intake on test performance.

**Results:**

OLP intake compared to no intervention was associated with improved test performance in those students with higher levels of test anxiety and those who adopted beneficial LS.

**Discussion:**

These findings provide preliminary evidence that psychological processes, such as anxiety or the application of cognitive strategies, modulate the effects of OLPs on cognitive performance in exam situations, framed within the context of self-efficacy. Further pre-registered, hypothesis-driven research is warranted, as harnessing these processes in the light of OLP applications could optimize students' well-being and maximize their academic success, including long-term potential for optimizing therapeutic outcomes in clinical settings.

## 1 Introduction

Cognitive performance is a central aspect of everyday functioning and is vital to success in education and work (Lövdén et al., 2020). Daily educational stressors such as deadlines or interpersonal conflicts, however, can impair cognitive performance, e.g., in university students in the preparation of and during testing situations. They affect not only self-reported academic performance (McBride and Greeson, 2023), but also objectively measurable cognitive outcomes such as short-term memory, attention (Vedhara et al., 2000), or course grades (Frazier et al., 2019).

Test anxiety, a common trait (Spielberger, 1995) that specifically occurs in challenging situations, can negatively impact academic performance (Richardson et al., 2012). It includes an emotional as well as a cognitive component such as worry or lack of confidence regarding upcoming testing situations. Motivational factors (e.g., self-efficacy) and learning strategies have also been found to predict academic performance (Richardson et al., 2012; Saks, 2024). Learning strategies refer to the use of cognitive strategies, such as organization or critical thinking, metacognitive strategies such as planning or monitoring, and resource-related strategies, such as time management and peer learning.

The subjective need to manage the impact of stressors on academic performance has led to a growing demand for cognitive enhancement through ‘smart drugs', e.g., methylphenidate and modafinil, among university students, despite the unclear or, at best, minimal benefits of these drugs and their significant harms (Schifano et al., 2022). Their positive impact may, in turn, substantially rely on placebo effects, which refer to the beneficial changes induced by expectation or prior experience (Carlino et al., 2014; Bingel, 2020). Indeed, placebo effects or positive expectations in the absence of active pharmacological treatment have been successfully shown to enhance various aspects of cognitive performance (Winkler and Hermann, 2019; Foroughi et al., 2016; Schwarz and Büchel, 2015; Parong et al., 2022; Colagiuri et al., 2011; Sinke et al., 2016; Benson et al., 2022), with significant improvement in subjective perception often not paralleled by objective improvement (Schwarz and Büchel, 2015; Winkler and Hermann, 2019).

Overcoming the ethical and legal limitations of deceptive placebos, open-label placebo (OLP) treatments, which are administered unconcealed and thus with the individual's full awareness, are allowing further expansion of placebo research (Kaptchuk, 2018). Recent meta-analyses reveal OLPs to be a promising treatment for subjective symptoms such as pain or measures of psychological well-being, both in clinical samples (Von Wernsdorff et al., 2021) and healthy volunteers (Spille et al., 2023). Furthermore, the specific effects of OLPs on cognitive performance and their predictors, such as test anxiety, has been given special attention over the past 5 years (Schaefer et al., 2019; Buergler et al., 2023; Schaefer and Enge, 2024; Hartmann et al., 2023; Kleine-Borgmann et al., 2021). OLPs have been shown to enhance cognitive performance within real-life testing situations, such as academic exams (Buergler et al., 2023) or driver's license tests (Schaefer and Enge, 2024). In contrast, they have shown no significant impact on experimental tasks like working memory or selective attention, which typically lack real-life consequences for the individual (Hartmann et al., 2023).

In a previous study in healthy medical students who were preparing for a central examination, we found that a 3-week OLP treatment had a positive impact on students' subjective well-being (i.e., stress, negative mood, fatigue and confusion), although the OLP treatment did not lead to higher exam scores compared to a no-treatment control group (Kleine-Borgmann et al., 2021). However, students in the OLP group who had a higher belief in the benefits of medication scored better on the exam (Kleine-Borgmann et al., 2021). It is thus reasonable to assume that cognitive and/or psychological processes may modulate the effects of OLPs on cognitive performance. Based on a subgroup of students from this previous study in whom learning strategies were assessed (Kleine-Borgmann et al., 2021), we aimed to conduct secondary, exploratory, and hypothesis-generating analyses on the effect of OLP intake on cognitive performance (i.e., the central examination result) and potential predictors of exam results, i.e., test anxiety and learning strategies. Potential additional effects of psychological distress measures, personality traits, pre-treatment expectations and biological sex were also explored, given their reported influences on placebo effects (Vambheim and Flaten, 2017; Bingel, 2020; Frisaldi et al., 2023). Therefore, we exploratively investigated: i) the interactions between OLP intake, test anxiety and learning strategies, and their effects on test performance in an academic examination and ii) the possible impact of psychological distress, personality traits, pre-treatment expectations, and biological sex on these interactions.

## 2 Methods

### 2.1 Study registration

Please note that the present study is an exploratory subgroup analysis of a recently published study. The original monocentric, single-blind randomized controlled, between-subjects study was pre-registered in the German Clinical Trials Register on December 20, 2017 (ID DRKS00013557; https://drks.de/search/de/trial/DRKS00013557). The main results have been published in Kleine-Borgmann et al. (2021).

### 2.2 Ethics statements

All study procedures were approved by the Ethics committee of the University Hospital Essen (17-7553-BO) and conducted in accordance with the Declaration of Helsinki (World Medical Association, 2013) and the principles of Good Clinical Practice (European Medicines Agency, 2016). Participants received financial compensation for their participation and were informed that they could withdraw from study participation at any time without giving reasons or having any disadvantages. There were no academic incentives for the participants.

### 2.3 Participants

Healthy medical students of the University Duisburg-Essen were recruited through flyers at regional universities and the University Hospital Essen, and via social media [see exact wording of the advertisement in Kleine-Borgmann et al. (2021)]. Medical students between 18 and 40 years who successfully registered for the central examination and provided voluntary written consent were eligible for the study. Exclusion criteria comprised a history of serious medical illness (especially diagnosed psychiatric illness or regular use of psychotropic substances), known allergies or intolerances to any of the ingredients of the placebo pills, ongoing participation in other studies, pregnancy or breastfeeding. Additionally, following data acquisition, participants with self-reported OLP compliance <70% were excluded in accordance with a per-protocol approach designed to ensure validity of the treatment effect and in line with our previous studies on OLP (Kleine-Borgmann et al., 2019) (see below).

Please note that the published main study included a sample of *N* = 154 participants for data analysis. However, not all of them filled out the learning strategy questionnaire, which was a prerequisite for this present exploratory analysis. A sample of *N* = 111 students completed all questionnaires required for the present study and were therefore eligible for data analysis. In addition, data from *n* = 7 participants had to be excluded (*n* = 1: withdrawal from exam participation; *n* = 6: participants from the OLP group with compliance <70%). The final subgroup sample therefore comprised *N* = 104 participants, 54 participants in the OLP group and 50 in the control group. Self-reported OLP compliance in this final sample was 93.65 ± 8.34%.

### 2.4 Study design

#### 2.4.1 Informed consent and randomization

As detailed in the main study, the general informed consent form was identical for all participants and included neutral, general information about placebo effects and the fact that they can influence cognitive performance. The concept of OLPs was also explained, alongside a short paragraph summarizing the results of recent OLP trials and a brief discussion of potential underlying mechanisms, which remain to be fully established—see Kleine-Borgmann et al. (2021), and specifically Supplementary material S1 for the exact wording and information provided prior to group assignment. After a baseline measurement, participants were randomized to the OLP or control group by a blinded investigator (single-blind) using a randomization algorithm implemented in RStudio (RStudio, version 1.2.5033, RStudio Team, RStudio: Integrated Development for R. RStudio, Inc., Boston, MA, USA). The OLP group received a 3-week OLP treatment, while the control group received no intervention.

**Figure 1:**
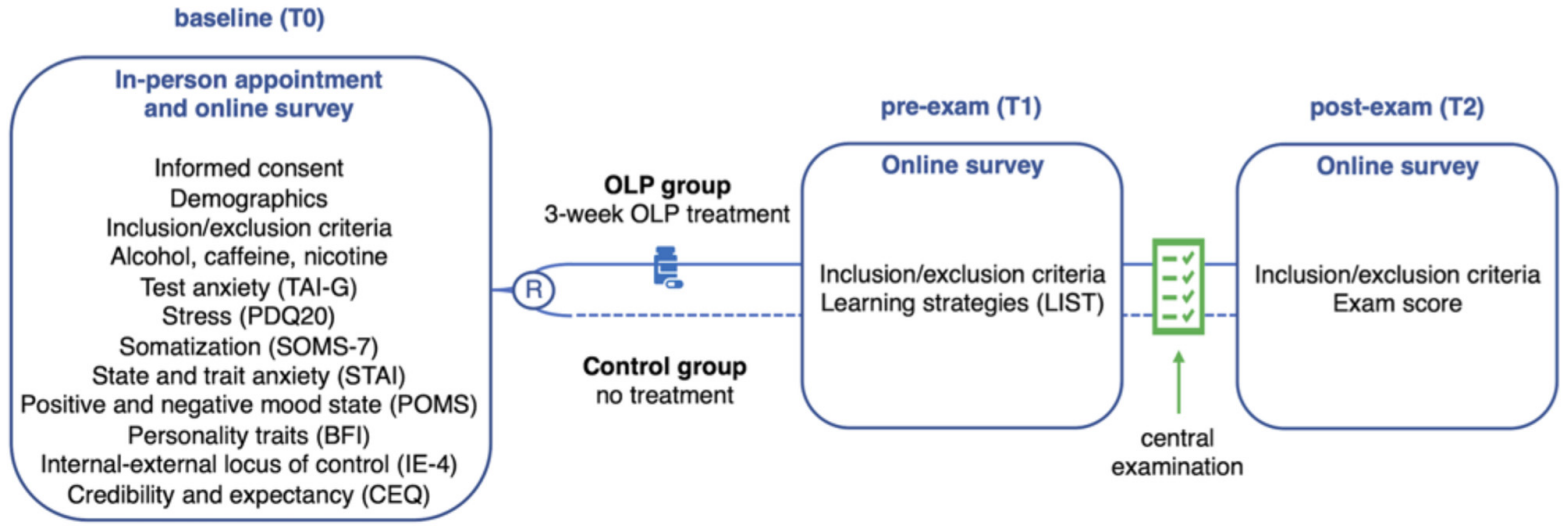
Study design. Assessments at: baseline (T0) prior to randomization **(R)** to either the open label placebo (OLP) group (i.e., 3-week OLP treatment, twice a day) or the control group (i.e., no treatment group); pre-exam (T1), i.e., 3 days prior to the exam; post-exam (T2), i.e., 60 days after the exam. Exam score (primary outcome) was assessed at T2 and calculated as percentage of correct answers in a standardized written central examination. The following standardized questionnaires were assessed at T0: German Test-Anxiety Inventory (TAI-G), Perceived Stress Questionnaire (PSQ20), Screening for Somatoform Disorders-7 (SOMS-7), State-Trait-Anxiety Inventory (STAI), Profile of Mood States (POMS), Big Five Inventory (BFI), Internal-External Locus of Control Short Scale-4 (IE-4), Credibility and Expectancy Questionnaire (CEQ). Learning strategies were assessed at T1 by the Inventory for Learning Strategies (LIST).

The study design (Figure 1) is described in detail in Kleine-Borgmann et al. (2021). The study consisted of one in-person appointment that included a self-administered online survey (T0), plus two online survey appointments (T1 and T2). Participants completed the German versions of the following questionnaires: Test Anxiety Inventory (TAI-G) (Hodapp, 1991; Hodapp and Benson, 1997; Harpell and Andrews, 2012), Perceived Stress Questionnaire (PSQ20) (Fliege et al., 2005), Screening of Somatoform Disorders-7 (SOMS-7) (Rief and Hiller, 2003), State-Trait-Anxiety Inventory (STAI) (Spielberger, 2010), Profile of Mood States (POMS) (McNair et al., 1992), Big Five Inventory (BFI) (Rammstedt and John, 2007), Internal-External Locus of Control Short Scale-4 (IE-4) (Nießen et al., 2022), and Credibility and Expectancy Questionnaire (CEQ) (Devilly and Borkovec, 2000). Pre-exam measures (T1, 3 days before the exam) included the completion of the Inventory for Learning Strategies (German: Inventar zur Erfassung von Lernstrategien im Studium, LIST) (Wild et al., 1992; Wild and Schiefele, 1994), which had not been analyzed in the main study (Kleine-Borgmann et al., 2021). Post-exam assessment (T2, 60 days after the exam) included documentation of the individual exam score and open-format feedback.

#### 2.4.2 Open-label placebos

All materials were handed over in a cardboard box by a blinded experimenter, who was instructed to avoid any communication about the contents and properties of the boxes. Participants were instructed not to open the cardboard box until they got home. OLPs were provided in a box containing 45 white-blue placebo pills (Zeebo Relief from Zeebo Effect, LLC, South Burlington, Vermont, USA) and a note emphasizing that the included pills contained no active treatment. As in previous OLP trials (Hartmann et al., 2023; Kleine-Borgmann et al., 2021, 2019; Carvalho et al., 2016; Hoenemeyer et al., 2018; Kaptchuk et al., 2010), participants in the OLP group were asked to take one placebo pill twice a day for a period of 3 weeks before the examination date. Participants in the control group received the same cardboard box, with filler materials having similar weight and producing similar accompanying noises, but no OLP pills. It also contained a note stating that the participant had been assigned to the control group and that no further action was required. For all participants in the OLP group the placebo intake ended on the day of the central examination.

### 2.5 Outcome measures

In addition to the primary outcome and covariates described below, the survey included: (i) standardized questionnaires to obtain demographic information and assess average alcohol, caffeine and nicotine consumption within a 7-day period at baseline; (ii) positive or negative (adverse) effects reported through the open-format response (“Have you noticed positive/negative effects of the open-label placebo application? If so, which?”) at the post-exam assessment.

#### 2.5.1 Primary outcome of the main study

The exam score of a standardized written central examination was acquired as the primary outcome. Medical students at the university Duisburg-Essen are required to take part in the bi-annual standardized exam with a multiple-choice response format, the so-called central examination (German: Zentrale Abschlussklausur). For all students, the exam is held over 2 days and, depending on the academic year, contains between 100–200 questions. Multiple academic subjects are tested in a short period of time, depending on the semester of each student. To ensure comparability of examination situations, the exam score was thus calculated by including only the academic subjects examined on the first day. Specifically, the exam score was calculated as the percentage of correct answers across all exam items.

#### 2.5.2 Exploratory covariates of the present study

The TAI-G is a validated and widely used inventory to measure self-reported test anxiety (Hodapp, 1991; Hodapp and Benson, 1997; Harpell and Andrews, 2012), which has been derived and further developed from the STAI (Spielberger, 2010). The TAI-G is a multidimensional measure consisting of four subscales: *worry, emotionality, lack of confidence*, and *interference*. *Worry* refers to the cognitive manifestation of concern about the consequences of failing, while *emotionality* captures the autonomic reactions that tend to occur under examination. *Lack of confidence* defines students' belief in their inability to perform well in an upcoming exam, while *interference* relates to the presence of thoughts interfering with on-task performance that are not a component of worry *per se* (Hodapp, 1991; Hodapp and Benson, 1997; Harpell and Andrews, 2012). For a view of all items, including the assignment to subscales, see Supplementary material S2 and Supplementary Table S1.

The LIST inventory is a slightly modified German translation of the Motivated Strategies for Learning Questionnaire (MSLQ) (Duncan and McKeachie, 2005). It uses three different scales to measure cognitive, metacognitive and resource-related learning strategies (from this point on referred to as LS) which, in turn, are divided into a total of eleven subscales (see Supplementary material S3 and Supplementary Table S2) (Wild et al., 1992; Wild and Schiefele, 1994).

*Cognitive LS* include those processes involved in the immediate absorption, processing, and storage of information. The following subscales build the cognitive LS scale: *organization, elaboration, critical thinking*, and *rehearsal*.

*Metacognitive LS* represent an important component of self-regulatory frameworks of learning and include planning the content and timing as well as the sequence of learning. This scale is also referred to in the literature as the “eleventh subscale” and accordingly does not divide into further subscales.

*Resource-related LS* aim to capture students' ability to manage the resources available to them. They include both external (i.e., *learning context, peer learning*, and *literature* subscales) and internal (i.e., *effort, attention*, and *time management* subscales) resources, which must be made available to achieve the learning goal.

As additional exploratory covariates, we examined biological sex together with questionnaires pertaining to: i) psychological distress measures, i.e., PSQ20 (*stress*), SOMS-7 *(somatic symptoms count and intensity of somatic symptoms)*, STAI-S (*state anxiety*), POMS (*total mood disturbance*); ii) characterization of personality traits, i.e., BFI (*neuroticism, extraversion, openness, conscientiousness, agreeableness*) and IE-4 (*internal locus* and *external locus of control*); iii) participants pre-treatment expectations, i.e., CEQ (*credibility* and *expectancy*).

### 2.6 Statistical analysis

Statistical analyses were performed with Rstudio (Rstudio, version 1.2.5033, Rstudio Team, Rstudio: Integrated Development for R. Rstudio, Inc., Boston, MA). In this exploratory analysis, however, effects were deemed potentially meaningful and replicable if their 95% confidence intervals (CIs) do not include zero and their effect size could at least be considered as small (partial eta squared $\eta_{p}^{2}$ ≥ 0.01). This approach aligns with recommended practices of reporting both effect size and confidence intervals to better assess estimation uncertainty (Schwab, 2015; Schünemann et al., 2019).

To test whether the OLP group and the control group differed at baseline (T0) and before taking the central examination (T1), demographic data and questionnaire scores were compared using Welch's two-sample t-tests or Pearson's Chi-square tests, as appropriate. Descriptive results are given as means with standard deviation or as frequencies in Table 1.

**Table 1:** Overview of group differences at baseline and pre-exam.

**Measures and subscales**	**Mean (SD) or frequency**		**N**	**95% CIs**	**t/Chi^2^**	***p***	**Effect size ($\eta_{\text{p}}^{2}$)**
	**OLP group**	**CTR group**	**OLP/CTR**				
**Baseline**							
Age	22.90 (2.74)	23.82 (2.78)	51/50	−0.17, 2.01	1.67	0.097	0.027
**Gender**							
Female	39	29	54/50	−0.32, 0.04	2.32	0.128	0.022
Male	15	21					
Body-mass-index (kg/m^2^)	21.42 (5.08)	20.85 (4.76)	54/50	−2.48, 1.35	−0.59	0.558	0.003
Alcohol consumption (g/day)	6.41 (7.31)	5.91 (6.48)	54/50	−3.19, 2.18	−0.38	0.708	0.001
Caffeine consumption (g/day)	5.90 (5.18)	7.42 (7.50)	54/50	−9.89, 40.7	1.21	0.229	0.014
Nicotine consumption (g/day)	0.19 (0.82)	0.11 (0.44)	54/50	−0.32, 0.18	−0.55	0.580	0.003
TAI-G	66.52 (11.29)	64.06 (12.60)	54/50	−7.13, 2.21	−1.04	0.299	0.01
Worry	24.67 (5.72)	21.64 (5.41)	54/50	−5.19, −0.86	−2.77	0.007	0.069
Emotionality	13.33 (3.80)	13.82 (4.29)	54/50	−1.09, 2.07	0.61	0.543	0.004
Interference	10.02 (2.74)	10.10 (3.33)	54/50	−1.11, 1.27	0.14	0.892	0
Lack of confidence	18.50 (2.35)	18.50 (2.42)	54/50	−0.93, 0.93	0.00	1.000	0
PDQ20	32.87 (16.74)	33.87 (16.17)	54/50	−5.41, 7.4	0.31	0.758	0.001
SOMS-7 symptom count	7.69 (5.44)	6.80 (5.83)	54/50	−3.08, 1.31	−0.80	0.426	0.006
SOMS-7 symptom intensity	11.74 (10.11)	10.18 (11.10)	54/50	−5.70, 2.58	−0.75	0.456	0.005
STAI-S, state anxiety	35.17 (8.94)	35.64 (7.83)	54/50	−2.79, 3.74	0.29	0.774	0.001
POMS	2.96 (17.59)	4.38 (19.23)	54/50	−5.77, 8.60	0.39	0.697	0.001
**BFI**							
Neuroticism	2.25 (0.35)	2.15 (0.26)	54/50	−0.22, 0.02	−1.72	0.088	0.027
Extraversion	1.79 (0.27)	1.75 (0.25)	54/50	−0.15, 0.06	−0.88	0.383	0.007
Openness	2.10 (0.27)	2.17 (0.33)	54/50	−0.04, 0.19	1.28	0.205	0.016
Conscientiousness	1.59 (0.23)	1.61 (0.26)	54/50	−0.07, 0.12	0.53	0.598	0.003
Agreeableness	2.07 (0.29)	2.01 (0.30)	54/50	−0.17, 0.05	−1.03	0.306	0.01
IE4 internal locus	4.33 (0.45)	4.33 (0.50)	54/50	−0.19, 0.18	−0.04	0.972	0
IE4 external locus	2.14 (0.69)	2.15 (0.68)	54/50	−0.25, 0.28	0.08	0.934	0
CEQ credibility	0.11 (2.58)	0.17 (2.59)	54/50	−0.95, 1.07	0.12	0.907	0
CEQ expectancy	0.05 (2.49)	0.08 (2.38)	54/50	−0.91, 0.99	0.08	0.937	0
**Pre-exam**							
LIST cognitive LS	12.36 (2.04)	12.42 (2.09)	54/50	−0.74, 0.86	0.15	0.884	0
Organization	3.27 (0.85)	3.23 (0.93)	54/49	−0.38, 0.32	−0.18	0.859	0
Elaboration	3.25 (0.64)	3.37 (0.64)	53/50	−0.12, 0.38	1.01	0.317	0.01
Critical thinking	2.32 (0.83)	2.59 (0.74)	54/49	−0.04, 0.58	1.74	0.085	0.028
Rehearsal	3.59 (0.68)	3.41 (0.74)	54/49	−0.46, 0.096	−1.30	0.197	0.016
LIST metacognitive LS	3.30 (0.61)	3.33 (0.56)	53/49	−0.2, 0.26	0.26	0.797	0.001
LIST resource LS	19.22 (3.01)	19.25 (2.36)	54/50	−1.02, 1.08	0.06	0.955	0
Effort	3.54 (0.57)	3.54 (0.47)	53/49	−0.21, 0.20	−0.04	0.968	0
Attention	3.03 (0.53)	3.11 (0.58)	54/48	−0.14, 0.30	0.73	0.468	0.005
Time management	3.11 (0.98)	3.13 (0.98)	52/50	−0.36, 0.40	0.1	0.920	0
Learning context	3.80 (0.71)	3.76 (0.71)	53/50	−0.32, 0.24	−0.30	0.765	0.001
Peer learning	2.69 (0.78)	2.51 (0.81)	54/49	−0.49, 0.13	−1.17	0.246	0.013
Literature	3.49 (0.95)	3.45 (0.76)	51/50	−0.38, 0.3	−0.23	0.815	0.001

*SD*, standard deviation; *N*, number; *t/Chi*^2^, Welch's two-sample *t*-test or Pearson's Chi-square test; *95% CIs, 95% Confidence Intervals*; $\eta_{p}^{2}$, partial eta squared; *p, p*-value; OLP, open-label placebo; CTR, control; kg/m^2^, a person's weight in kilograms/height in meters squared; g/day, grams per day; TAI-G, German Test Anxiety Inventory; PDQ20, Perceived Stress Questionnaire; SOMS-7, Screening of Somatoform Disorders-7; STAI-S, State and Trait Anxiety Inventory-State; POMS, Profile of Mood State; BFI, Big Five Inventory; IE4, Internal–External Locus of Control Short Scale−4; CEQ, Credibility and Expectancy Questionnaire; LIST, Inventory for Learning Strategies (German: Inventar zur Erfassung von Lernstrategien im Studium questionnaire); LS, learning strategies.

For the exploratory analyses, we followed a sequential approach: First, as in our publication of the main study (Kleine-Borgmann et al., 2021), the OLP treatment effect on the primary outcome was tested in our smaller sample using a general linear model (GLM) comparing exam scores between groups (mean difference between OLP group and control group *M*_*diff*_; between-subjects factor), with *exam score* as the dependent variable and *group* as the independent variable.

In a second step, potential interactions between *test anxiety* (i.e., TAI-G) and OLP intake in their effect on the exam score were calculated using a GLM, with *exam score* as the dependent variable and *group* as well as *test anxiety* as independent variables. As a follow-up analysis focusing on the observed effects of test anxiety, we examined OLP effects on test performance in highly anxious students. Following the approach of Dutke and Stöber (2001) as well as Stöber and Esser (2001), we divided the TAI-G scores into tertiles (i.e., < 33.3%, ≥ 33.3% and < 66.6.%, ≥ 66.6%; range of 0–59, 60–68, and 69–108, respectively). The range of TAI-G scores for students with higher test anxiety in the control group (*N* = 14) was 70–108 and 69–100 for students in the OLP group (*N* = 22). Then, we tested for group differences in exam scores in those participants with test anxiety levels in the upper third of the distribution.

Third, in three separate GLMs for each of the three LS scales, the potential interaction between LS (i.e., LIST) and OLP intake in their effects on the exam score was calculated in the same way.

When interaction effects involving test anxiety or LS showed 95% CIs that did not include zero and their effect size could be considered as at least small, the individual subscales of the respective questionnaires were further explored as potential covariates to identify which components most contributed to the observed effects.

Further exploratory analyses tested interactions between: (i) *group, test anxiety*, and *LS*; (ii) *group, text anxiety*, and, respectively, *biological sex, PSQ20, SOMS-7, STAI-S, POMS, BFI, IE-4, CEQ*; and (iii) *group, LS*, and, respectively, *biological sex, PSQ20, SOMS-7, STAI-S, POMS, BFI, IE-4, CEQ*. For these, too, interactions with entirely positive 95% CIs and $\eta_{p}^{2}$ ≥ 0.01 were followed up with subscale analyses.

Because these analyses were secondary, not pre-registered, and not powered for formal hypothesis testing, no corrections for multiple comparisons were applied, in line with the exploratory and hypothesis-generating nature of the study. Effect sizes were calculated using $\eta_{p}^{2}$, interpreted as negligible ($\eta_{p}^{2}$ < 0.01), small (0.01 ≤ $\eta_{p}^{2}$ < 0.06), moderate (0.06 ≤ $\eta_{p}^{2}$ < 0.14), and large ($\eta_{p}^{2}$ ≥ 0.14) (Cohen, 1988). GLM results include unstandardized beta coefficients (ß) with their standard errors (*SE*) and 95% confidence intervals (CIs).

## 3 Results

Interaction effect results that were negligible and imprecisely estimated (η_*p*_^2^ < 0.01, 95% CI including zero), along with a few exceptions of small but imprecisely estimated effects (0.01 ≤ $\eta_{p}^{2}$ < 0.06, 95% CI including zero), are provided as Supplementary material, specifically in Supplementary Tables S3–S7.

OLP group and control group were comparable regarding all measures investigated at T0 and T1 except for the *worry* TAI-G subscale (OLP > CTR), as shown in Table 1. As reported previously (Kleine-Borgmann et al., 2021), OLP intake compared to no treatment did not lead to generally higher exam scores across the sample [M_*diff*_ = 0.52 ± 1.61, 95% CI (−2.63, 3.66), *t*_(103)_ = 0.32, *p* =0.749, $\eta_{p}^{2}$ = 0.001].

### 3.1 Interaction effects between group and test anxiety on exam score

**Figure 2:**
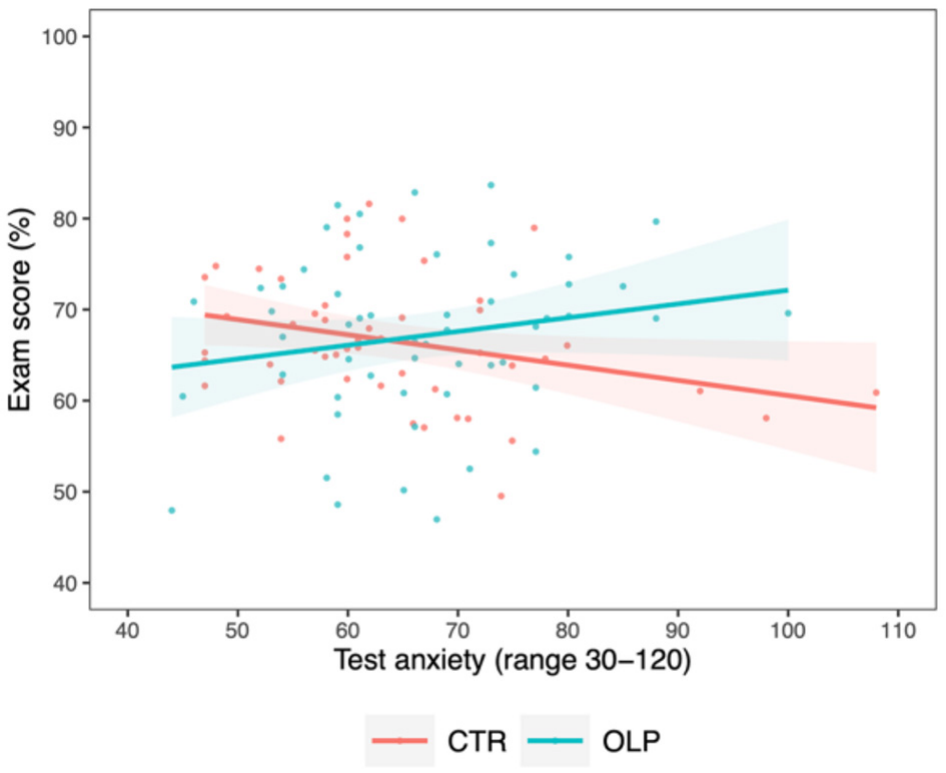
Correlation between exam score and test anxiety for the control group (CTR, red) and the open label placebo (OLP) group (blue), respectively, including individual data points and 95% confidence intervals (shaded bands). Compared to the control group, a positive interaction between test anxiety and OLP intake in their effects on the exam score was present, pointing toward a beneficial effect of OLP intake on exam performance in students with higher test anxiety.

The exploratory analysis revealed a potentially meaningful interaction of small effect size between the factors *group* and *test anxiety* on *exam score* [*M*_*diff*_ = 0.32 ± 0.13, 95% CI (0.06, 0.58), *t*_(103)_ = 2.38, *p* =0.019, $\eta_{p}^{2}$ = 0.053]. Students in the control group with higher levels of test anxiety showed lower exam scores. Interestingly, students in the OLP group who had reported higher levels of test anxiety at baseline scored better on the central examination (Figure 2) pointing toward a beneficial effect of OLP intake when suffering from test anxiety. This result was further confirmed by the subgroup analysis of n = 36 students with high levels of test anxiety. Here, OLP intake compared to no treatment was associated with higher exam scores [*M*_*diff*_ = 5.71 ± 2.54, 95% CI (0.72, 10.7), *t*_(35)_ = 2.24, *p* =0.031, $\eta_{p}^{2}$ = 0.129], reflecting a moderate effect. The mean exam score was 62.92 (*SD* = 7.35) in the control group and 68.63 (*SD* = 7.50) in the OLP group.

Following up on these findings, we explored potential differences in the influence of specific sub-components of test anxiety. Here, further small interaction effects emerged between *group* and the TAI-G subscales *worry* [*M*_*diff*_ = 0.64 ± 0.28, 95% CI (0.08, 1.20), *t*_(103)_ = 2.24, *p* =0.028, $\eta_{p}^{2}$ = 0.048] and *emotionality* [*M*_*diff*_ = 0.82 ± 0.40, 95% CI (0.04, 1.60), *t*_(103)_ = 2.05, *p* =0.043, $\eta_{p}^{2}$ = 0.040; see also Supplementary Table S3]. In detail, students in the OLP group with higher levels of test anxiety domains specific for *worry* and *emotionality* performed better on the central examination compared to students in the control group. However, given that OLP and control groups already differed in *worry* subscale at baseline (Table 1), caution must be applied to the interpretation of which aspects of the test anxiety constructs were driving the observed effects. Importantly, the analysis including *emotionality* as a covariate still showed an interaction with small effect size when controlling for group differences in *worry* [*M*_*diff*_ = 0.78 ± 0.39, 95% CI (0.01, 1.56), *t*_(103)_ = 1.99, *p* =0.049, $\eta_{p}^{2}$ = 0.038].

### 3.2 Interaction effects between group and learning strategies on exam score

**Figure 3:**
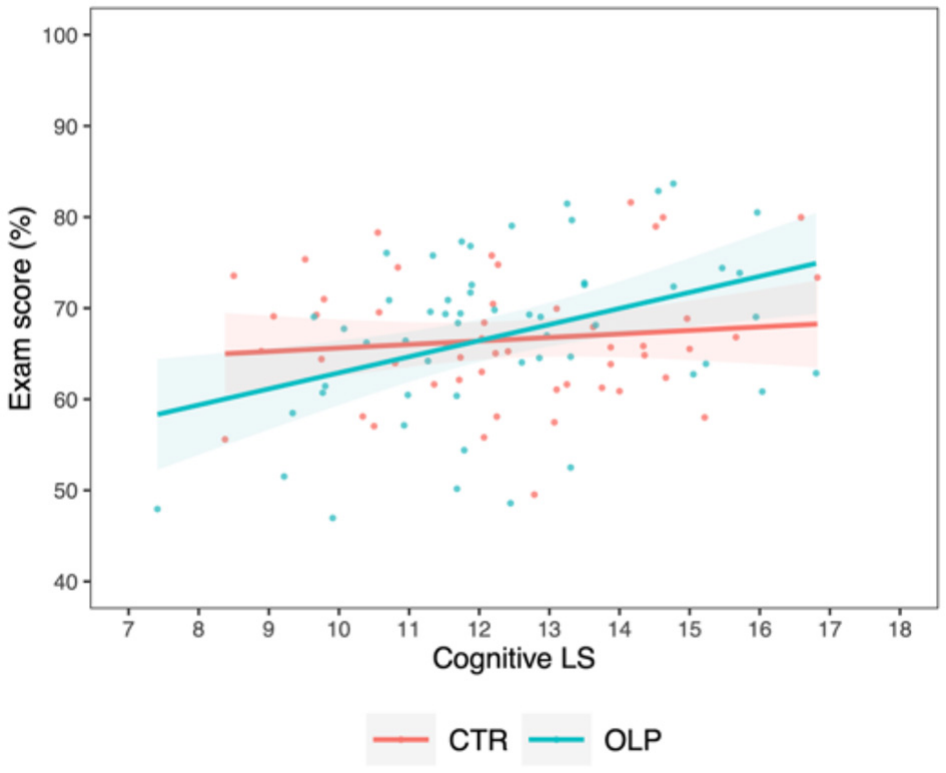
Correlation between exam score and cognitive learning strategies for the control group (CTR, red) and the open label placebo (OLP) group (blue), respectively, including individual data points and 95% confidence intervals (shaded bands). Compared to the control group, students in the OLP group appeared to benefit more from the use of cognitive LS in terms of exam performance.

When testing for effects of LS and OLP intake on exam score, we observed an interaction with small effect size between the factors *group* and *cognitive LS* on *exam score* [*M*_*diff*_ = 1.38 ± 0.75, 95% CI (−0.09, 2.85), *t*_(103)_ = 1.84, *p* =0.069, $\eta_{p}^{2}$ = 0.033]. In particular, students in the OLP group using more cognitive LS tended toward having higher exam scores compared to those in the control group (Figure 3).

**Figure 4:**
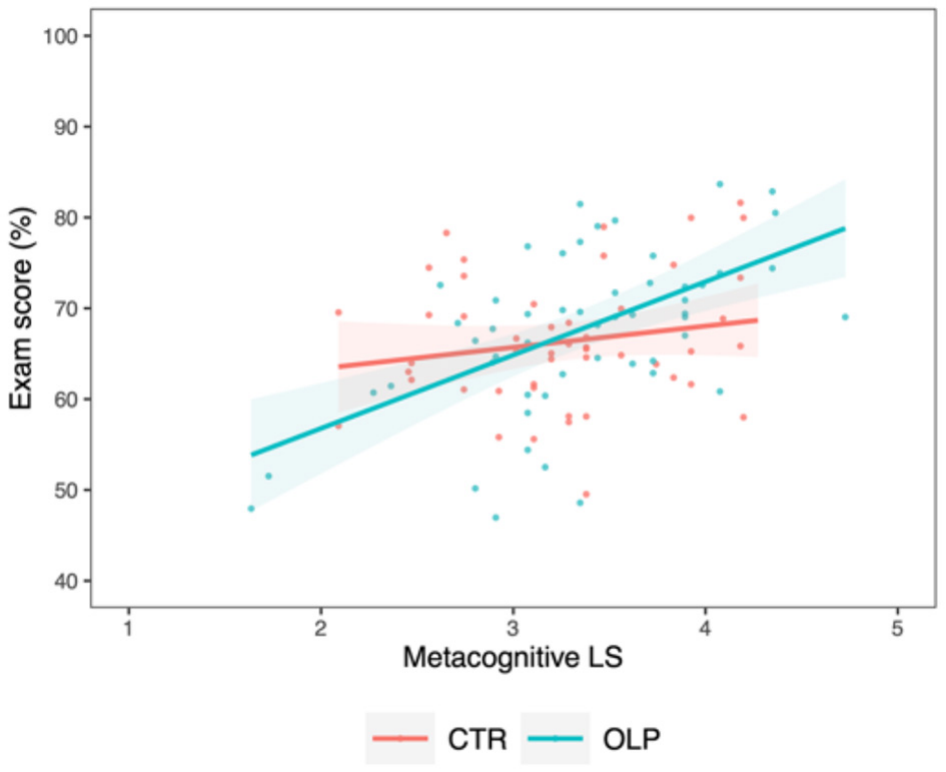
Correlation between exam score and metacognitive learning strategies for the control group (CTR, red) and the open label placebo (OLP) group (blue), respectively, including individual data points and 95% confidence intervals (shaded bands). Compared to the control group, a positive interaction between the use of metacognitive LS and OLP intake in their effects on the exam score was present, suggesting an advantageous effect of OLP intake on the use of metacognitive LS for examination success.

Further, students in the OLP group applying more metacognitive LS achieved better exam scores than students in the control group, as indicated by a stronger positive interaction with a small effect size between the usage of *metacognitive LS* and OLP intake on *exam score* [*M*_*diff*_ = 5.73 ± 2.53, 95% CI (0.76, 10.7), *t*_(103)_ = 2.26, *p* =0.026, $\eta_{p}^{2}$ = 0.050, Figure 4].

**Figure 5:**
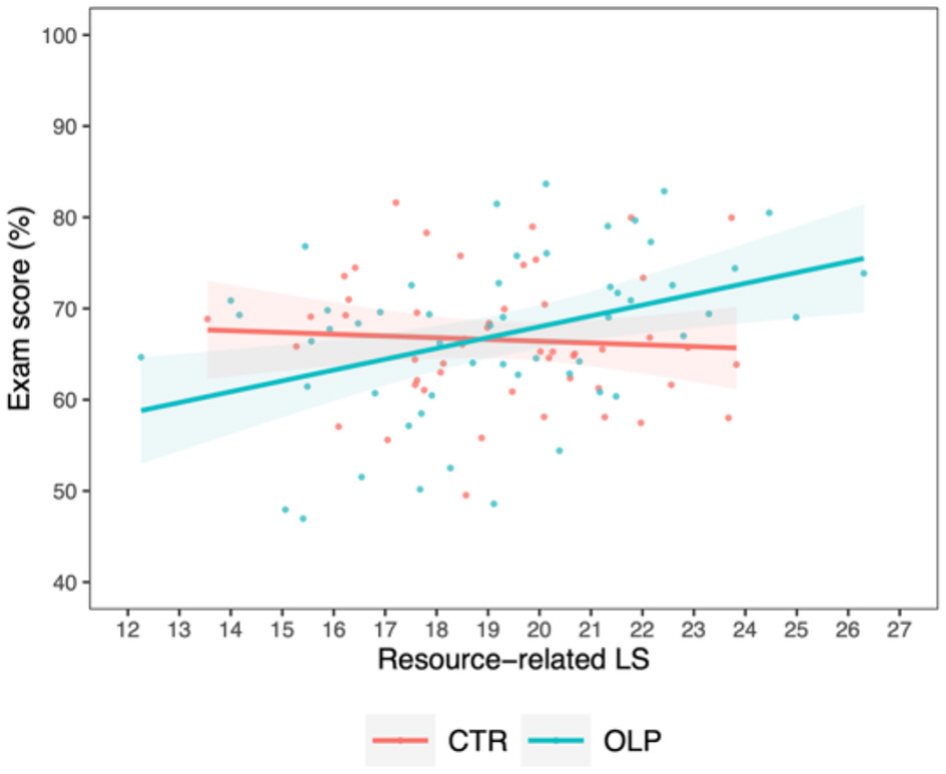
Correlation between exam score and resource-related learning strategies for the control group (CTR, red) and the open label placebo (OLP) group (blue), respectively, including individual data points and 95% confidence intervals (shaded bands). Compared to the control group, we observed a positive interaction between the use of resource-related LS and OLP intake on the exam score, suggesting an advantageous effect of OLP intake on the use of resource-related LS.

A potentially meaningful and replicable interaction with a small effect size between the factors *group* and *resource-related LS* on *exam score* was also observed [*M*_*diff*_ = 1.38 ± 0.59, 95% CI (0.22, 2.54), *t*_(103)_ = 2.32, *p* =0.022, $\eta_{p}^{2}$ = 0.051, Figure 5]. Unlike the control group, students in the OLP group using more resource-related LS yielded higher exam scores.

**Figure 6:**
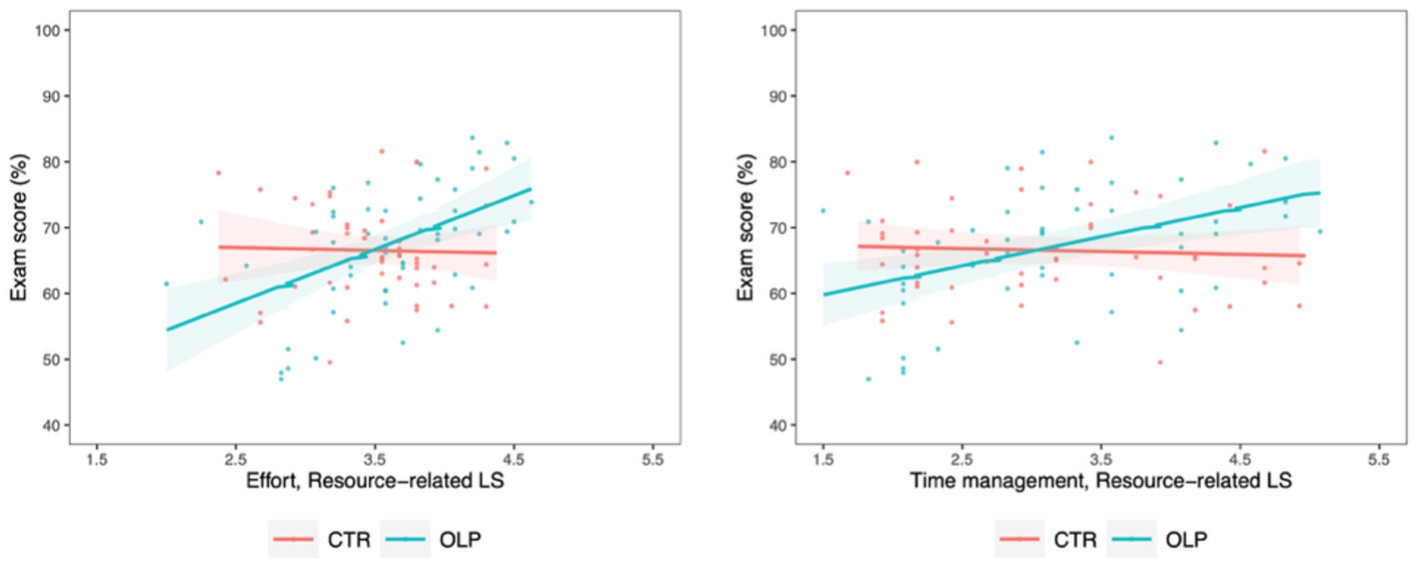
Correlation between exam score and resource-related learning strategies [i.e., effort **(left)** and time management **(right)**] for the control group (CTR, red) and the open label placebo (OLP) group (blue), respectively, including individual data points and 95%confidence intervals (shaded bands). Compared to the control group, we observed a positive interaction between the use of effort/time management strategies and OLP intake on the exam score, suggesting that these factors may be key determinants of the overall effects observed for resource-related LS.

Investigating potential differences in the above described effect of *resource-related LS* for individual components of this type of LS separately, analyses revealed that both *effort* and *time management*, and thus two internal resources, seem to contribute to the observed interaction effects [effort: *M*_*diff*_ = 8.63 ± 2.97, 95% CI (2.80, 14.46), *t*_(103)_ = 2.90, *p* =0.005, $\eta_{p}^{2}$ = 0.079; time management: *M*_*diff*_ = 4.56 ± 1.60, 95% CI (1.42, 7.70), *t*_(103)_ = 2.85, *p* =0.005, $\eta_{p}^{2}$ = 0.076], which were both moderate effects (Figure 6 and Supplementary Table S4).

### 3.3 Additional analyses

In terms of their influence on students' exam scores, negligible and imprecisely estimated interaction effects were found between *group, test anxiety* and, respectively, *cognitive LS, metacognitive LS, resource-related LS* (see Supplementary Table S5).

Further, interaction effects between *group, test anxiety*, and any other *additional exploratory covariate* on *exam score* were negligible, including those involving biological sex, with a few exceptions of small but nevertheless imprecisely estimated effects (see Supplementary Table S6).

Among the interactions between *group, LS*, and any other *additional exploratory covariate* on *exam score*, we observed two potentially meaningful three-way interactions of small effects size between the factors *group, cognitive LS*, and *somatic symptoms* {i.e., SOMS-7: *somatic symptoms count [M*_*diff*_ = 0.37 ± 0.16, 95% CI (0.06, 0.69), *t*_(103)_ = 2.34, *p* =0.021, $\eta_{p}^{2}$ = 0.054]; SOMS-7: *intensity of somatic symptoms* [*M*_*diff*_ = 0.18 ± 0.09, 95% CI (0.01, 0.35), *t*_(103)_ = 2.11, *p* =0.038, $\eta_{p}^{2}$ = 0.044]}. In the OLP group, higher use of cognitive learning strategies was still correlated with higher exam scores despite higher somatic symptom scores (see Supplementary Figure S1). No other potentially meaningful interaction effects were found (see Supplementary Table S7).

As part of the open format feedback, one participant in the OLP group reported flatulence as a treatment side effect. Three students provided negative feedback: two participants reported stress about forgetting to take the capsules regularly and one highlighted the inconvenience of the capsule intake twice daily. Seven students gave positive feedback including “that the OLP helped them structure their daily routine”, “increased their motivation”, had an “overall encouraging effect” (Supplementary material S4).

## 4 Discussion

This secondary analysis of a randomized controlled trial explored potential effects of a 3-week OLP treatment, test anxiety and learning strategies on academic test performance in *N* = 104 medical students. Our hypothesis-generating findings suggest that, compared to the control group, OLP intake may be associated with improved test performance in students with higher levels of test anxiety and in those who adopt beneficial learning strategies. These observations may indicate that the effects of OLP intake on cognitive performance are potentially influenced by psychological processes such as anxiety or the application of cognitive strategies.

Importantly, the present findings—derived from a smaller sample of the main study publication (Kleine-Borgmann et al., 2021)—are consistent with earlier results indicating that OLP intake does not generally lead to higher exam scores compared with no treatment. This finding is consistent with limitations of expectation effects emerging from both deceptive placebos (Schwarz and Büchel, 2015; Winkler and Hermann, 2019) and OLPs (Von Wernsdorff et al., 2021; Hartmann et al., 2023; Spille et al., 2023), whereby subjective gains may not necessarily be reflected in objective performance parameters. However, this contrasts with Buergler et al. (2023), who reported significantly improved test performance in intervention groups (using an imaginary pill intervention or an OLP intervention) compared to the control group. These divergent results could arise from differences in sample characteristics (as only healthy students with self-reported test anxiety were included in this study), testing format, and expectation levels influenced by design (i.e., higher probability of being assigned to an experimental group; (Buergler et al., 2023).

Our present analysis suggests that students with higher levels of test anxiety may have benefited more from the OLP treatment, as reflected in higher central examination performance. Specifically, compared to the control group, the genuine negative effect of test anxiety not only decreased in the OLP group, but test performance was even higher (with a small effect size). Test anxiety, and especially its emotional component (i.e., emotionality), may thus be particularly susceptible to OLP treatment, pointing to potential applications for optimizing therapeutic outcomes in clinical settings of anxiety treatment. However, results regarding specific influences of cognitive component of test anxiety should be interpreted cautiously considering a baseline group difference in the worry subscale.

Students with higher level of test anxiety have been shown to have difficulty retrieving information. For this reason, they perform better on multiple-choice tests in which they are required to recognize the correct answer rather than to remember or actively reproduce it (Naveh-Benjamin et al., 1981). Therefore, the central multiple-choice examination taken by the participants in our study may have elicited a reduced level or a rather small effect of test anxiety on exam results in the predisposed students, possibly contributing to the observed small effect sizes.

Despite previous literature suggesting that placebo effects and anxiety responses may be modulated by biological sex (Vambheim and Flaten, 2017; Frisaldi et al., 2023), our data did not reveal any sex-related effects. This may reflect limitations in sample size or differences in the specific tools used to measure anxiety.

What psychological mechanisms underlie this interplay of OLP effects and test anxiety is currently unclear. Our previous study showed that students with stronger beliefs in the effect of medication benefited more from OLP treatment with regard to test performance (Kleine-Borgmann et al., 2021), which can be seen as another indicator for the influence of psychological processes on OLP effects, such as the expectation toward a treatment. It might also be possible that our study participants associated placebo pills with ‘smart drugs', whose utilization to potentially increase concentration ability, learning capacity, and academic performance is growing rapidly (Greely et al., 2008; Carton et al., 2018; Schifano et al., 2022). This assumption may have been prompted by the fact that students were informed before study participation that placebo effects can enhance cognitive performance (Colagiuri et al., 2011; Schwarz and Büchel, 2015; Foroughi et al., 2016; Sinke et al., 2016, 2017; Winkler and Hermann, 2019; Parong et al., 2022). In addition, placebo effects in our study may have been triggered and potentiated by students' self-efficacy, which refers to both their expectancy for success (which is specific to task performance) and their belief to be able to fulfill academic tasks and successfully study materials (Duncan and McKeachie, 2005; Richardson et al., 2012; Hayat et al., 2020). In fact, students with higher self-efficacy expectations have been shown to perform better in tasks than those with lower self-efficacy expectations (Richardson et al., 2012). Therefore and according to previous literature (Schaefer et al., 2019; Buergler et al., 2023), the level of test anxiety might have decreased in students assigned to the OLP group. This could then have promoted an increase in students' levels of self-efficacy and, consequently, in their well-being and test performance (Richardson et al., 2012). However, this idea could generate hypotheses for future studies using longitudinal tracking of both test anxiety and self-efficacy (Buergler et al., 2023; Emadi Andani et al., 2024).

We observed potentially meaningful positive interactions of small effect size between OLP treatment and both metacognitive and resource-related LS, whereas the interaction with cognitive LS showed a similar effect size but with some uncertainty around the estimate. In particular, effort and time management subscales of resource-related LS exhibited moderate effect sizes, suggesting they are key determinants of the overall effects observed for this LS domain. Considering the multiple-choice testing format, volume of learning materials and the brevity of the central examination, it seems understandable that precisely effort and time management were the most beneficial LS. This observation is further in line with results from Fabry and Giesler (2007), namely that effort and attention have been found to be particularly relevant LS for medical students.

Although not represented in our sample, the effects of applied LS, test anxiety and self-efficacy have been shown to be directly connected. Specifically, Duncan and McKeachie (2005) reported that students with positive motivational beliefs such as high self-efficacy and task value, and with lower levels of test anxiety, tend to engage in deep-processing strategies such as elaboration and organization and seek to control of their cognition and behavior through the use of metacognitive planning, monitoring, and regulating strategies. These students, compared to those with less adaptive motivational beliefs, are more likely to perform better on assignments, exams and courses, as well as in their overall course grade (Duncan and McKeachie, 2005; Hayat et al., 2020).

It can also be discussed whether the ritual of undergoing OLP treatment may in itself be interpreted as a type of LS. This would mean extending the classical definition of LS to include positive expectation and experience effects. In line with that, when answering open-format questions 60 days after the exam, some students reported that the OLPs helped them structure their daily routines, increased their motivation, and had an overall encouraging effect—all consistent with increased self-efficacy. At the same time, as reported in Hartmann et al.'s (2023) study on the effects of OLPs on cognitive abilities in an experimental task, some students reported dissatisfaction with group allocation to the OLP group, potentially due to the additional effort they had to invest by taking the OLP capsules, which was not required of the control group. This cost consideration should thus be considered when planning and interpreting OLP studies.

In future studies, systematic recording of the time course of students' expectations (e.g., “How do you expect the OLP intake to influence your preparation for and performance in the exam?”), their experience in taking OLPs (e.g., “How did taking OLPs affect your exam preparation and performance?”), and the exact circumstances under which the placebo pills were taken (e.g., “Have you developed rituals associated with taking OLP?”) would therefore be relevant to understand and characterize the (un)conscious mechanisms underlying OLPs effects. Systematic collection of information also related to different testing situations, life circumstances, personality traits, as well as fluctuations of test anxiety and self-efficacy would provide insights into which students may benefit most from OLP treatments. Furthermore, the LIST-questionnaire was administered only once, i.e., just before the central examination, preventing the possibility of recording potentially different temporal dynamics of LS between the OLP and control groups during the treatment phase. In future studies, therefore, it would be important to quantify changes in LS over the course of OLP treatment.

Finally, potentially meaningful interactions between OLP intake, cognitive LS, and somatic symptoms emerged, with small effect sizes, suggesting a possible buffering effect of LS and OLP on somatic complaints and a potential contribution to well-being or self-efficacy.

Overall, interaction effects sizes ranged from small to moderate, ($\eta_{p}^{2^{\prime }}$s = 0.033 to 0.079).

We acknowledge the ethical considerations of using OLPs to address anxiety in healthy medical students (Spielberger, 1995; Kleine-Borgmann et al., 2021; Buergler et al., 2023). While OLPs are generally regarded as safe, their use in non-clinical populations carries the potential risk of overmedicalization, whereby normal variations in stress or performance anxiety could be inappropriately framed as requiring intervention. Careful consideration of context, necessity, and proportionality, such as differences between state anxiety, being anxious during exams and clinically relevant anxiety disorders, is therefore essential.

Our findings should be interpreted considering several limitations. First, as a secondary, exploratory analysis not preregistered or included in the original protocol, our study lacks sufficient statistical power to formally test hypotheses. Second, the per-protocol approach, which allowed us to highlight the maximum potential effect of the OLP treatment when taken as intended, does not provide results for participants with low compliance. Third, the absence of a direct measure of self-efficacy and the lack of longitudinal assessment of both test anxiety and LS limit our ability to fully understand the mechanisms underlying the observed effects. Finally, generalizability is limited, as medical students might be especially susceptible (Lim and Seet, 2007; Arnold et al., 2020) or, conversely, skeptical of placebo effects (Blackwell et al., 1972). Moreover, our recruitment via public call may have attracted students with a positive attitude toward OLPs.

## 5 Conclusion

This study suggests that OLP intake compared to no intervention may improve test performance in students with higher levels of test anxiety and those who adopted beneficial LS, supporting the idea of a crucial role of psychological processes in modulating the effects of OLPs on cognitive performance in exam situations. While these results are promising, they should be interpreted cautiously, given the exploratory and observational nature of the analysis, which limits causal interpretation. Introducing OLP treatment into the university or even school context as support for students while preparing for exams could still be a promising avenue but warrants further investigation. When combined with an adequate education about the possibilities and limitations of OLPs, their administration might offer students a personal success experience by increasing awareness of their internal and external resources, enhancing self-efficacy, optimizing well-being, and maximizing academic success. Nevertheless, the observed associations require confirmation in pre-registered, hypothesis-driven studies, which could also pave the way for exploring their potential application in clinical contexts.

## Data Availability

The datasets presented in this study can be found in online repositories. The names of the repository/repositories and accession number(s) can be found in the article/Supplementary material.
