# Supplementary material for: The association between test anxiety, learning strategies, and open-label placebo effects on academic test performance: a secondary analysis of a randomized controlled trial
Source: Front Psychol. 2025 Oct 9;16:1529056. doi: 10.3389/fpsyg.2025.1529056 (PMC12545060; doi:10.3389/fpsyg.2025.1529056)
Supplement: Supplementary file 1 [file Supplementary_file_1.docx]

**Supplementary information**

**Supplementary information S1**

*Excerpt of the informed consent explaining the rationale (full wording, translated from German)*

“Meta-analyses confirm that placebo treatments (inert drug missing an active ingredient) can have significant and clinically relevant effects on various conditions. Astonishingly, in conditions of acute and chronic pain and depression, for example, it has been shown that these positive effects also occur when the patient is aware of being treated with a placebo (so-called open-label placebo treatment (OLP)). The underlying mechanisms of this effect are not yet known. In particular, evolutionary conditioning processes but also psycho-neurobiological mechanisms of increased self-regulation/self-efficacy are discussed. Recent studies show that cognitive functions and well-being can be influenced by the placebo effect, especially in test situations. So far it is unknown whether open-label placebos can also influence cognitive performance and well-being, and which mechanisms are involved. This will be further investigated in this study. The test performance in the written semi-annual examination will be the objective criterion of cognitive performance in this study.”

*Information handed out at randomization*

Allocation to the OLP group: “Dear participant, you have been assigned to the group receiving a placebo application. Enclosed you will find the placebo capsules. Please open the container and discard the small bag that is intended to keep the capsules dry. Please take one capsule in the morning and one capsule in the evening at the beginning of the test period (in total 2 capsules per day) for 21 days. Please find the beginning’s date of the test period labeled on the container. We will also send you an e-mail reminder. Please make sure that you take the capsules regularly. Please store the container at room air and dry atmosphere. Please note the following when contacting us: The group allocation is blinded, so that your contact person does not know your group allocation and should only know it in case of an emergency. Many thanks for your cooperation!”

Allocation to the control group: “Dear participant, you have been assigned to the observation group. You will not receive placebo capsules during this study. Please find the beginning’s date of the test period labeled on the container. We will also send you an e-mail reminder. There is no need for further action after receiving the e-mail.”

**Supplementary information S2**

TAI-G questionnaire includes 4 subscales (worry, emotionality, lack of confidence, interference) which, in turn, are composed of 6-10 items each, for a total of 30 items. Each item is measured using a 4-point Likert scale, and the values of the lack of confidence subscale are inverted. Total scores and subscales demonstrate alpha coefficients ranging from 0.79 to 0.94^1^. The following are the items from the English version of the TAI-G questionnaire as reported by Harpell and Andrews (2012)^2^.

**Supplementary Table S1.** The TAI-G subscales, item assignment and examples of items

| **Subscale** | **Component** | **Item number** | **Example items** |
| --- | --- | --- | --- |
| Lack of confidence | Cognitive component | 1 8 13 20 26 29 | I am confident about my performance.  I think that I will succeed. |
| Emotionality | Emotional component | 3 7 12 16 18 22 25 28 | I get “butterflies”.  I feel anxious.  I feel overwhelmed. |
| Worry | Cognitive component | 2 4 6 9 10 14 17 21 23 27 | I think about how important the examination is for me.  I am thinking about the consequences of failing. |
| Interference | Cognitive component | 5 11 15 19 24 30 | Distracting thoughts keep “popping” into my head.  I forget things because I am too preoccupied with my personal problems. |

**Supplementary information S3.**

LIST inventory includes 3 scales — cognitive, metacognitive and resource-related strategies —and 11 subscales. The latter, in turn, are composed of 4-11 items each, for a total of 77 items. Each item is measured using a 5-point-Likert scale with the following anchors: ‘very rarely’, ‘rarely’, ‘sometimes’, ‘often’, ‘very often’. The internal consistency of the overall scale, assessed by Cronbach’s α, is between 0.75 and 0.91^3,4^.

The LIST is a slightly modified German translation of the Motivated Strategies for Learning Questionnaire (MSLQ)^5^. As the LIST has not yet been formally validated in the English language, we here report the example items from MSLQ and then provide the German-to-English translation done by our research team for the item examples related to the two sub-scales found exclusively in the LIST (i.e., *attention* and *literature)*.

**Supplementary Table S2** The LIST subscales, item assignment and examples of items

| **Scale** | **Subscale** | **Item numbers** | **Example items** |
| --- | --- | --- | --- |
| Cognitive  learning strategies | Organization | 1 14 25 36 47 56 65 72 | When I study the readings for this course, I outline the material to help me organize my thoughts. |
|  | Elaboration | 2 17 28 39 49 58 67 73 | When I study for this class, I pull together information from different sources, such as lectures, readings, and discussions. |
|  | Critical  thinking | 3 18 29 40 50 59 68 74 | I often find myself questioning things I hear or read in this course to decide if I find them convincing. |
|  | Rehearsal | 4 19 30 41 51 60 69 | When I study for this class, I practice saying the material to myself over and over. |
| Metacognitive  learning strategies | / | 5 6 13 20 31 42 52 61 70 75 77 | When reading for this course, I make up questions to help focus my reading.  Before I study new course material thoroughly, I often skim it to see how it is organized. |
| Resource-related  learning strategies | Effort | 9 21 32 43 53 62 71 76 | I work hard to do well in this class even if I don’t like what we are doing. |
|  | Attention* | 10 22 33 44 54 63 | I am not focused when I study. |
|  | Time  management | 11 23 34 45 | I make sure that I keep up with the weekly readings  and assignments for this course. |
|  | Learning  context | 12 24 35 46 55 64 | I usually study in a place when I can concentrate on my course work. |
|  | Peer learning | 7 15 26 37 48 57 66 | When studying for this course, I often try to explain the material to a classmate or a friend. |
|  | Literature* | 8 16 27 38 | I search for further reading if a particular content is not completely clear to me. |

Note. * Subscales exclusively present in the LIST inventory.

**Supplementary information S4** Open-ended reports voluntarily provided by some students during the post-exam assessment.

*Positive reporting*

- “Well-being because it became a kind of little ritual.”
- “It reminded me to study.”
- “It felt like having an extra little tool at my disposal and, on some days, I was a little more motivated.”
- “Fun taking the tablets.”
- “Structured work, fewer motivation problems, greater work performance.”
- “It lightens the mood before studying when you say to yourself: now I'll take my placebo and then I'll be able to study better.”
- “The motivation to study was slightly higher after taking it than in the previous semester. Taking it gave me courage in a way, because ‘nothing can go wrong, I'm taking placebo pills.”

*Side effects*

- “Severe flatulence, much to the discomfort of my roommates. Otherwise, none.”

Negative reporting

- “Having to think about it every day.”
- “Taking tablets twice a day became annoying at some point. You always had to remember to take them and (at least for me) you always had to drink water with them, which then had to be ready at all times.”
- “Stressed about forgetting the placebo.”

**Supplementary Table S3** Interaction effects between group and test anxiety on exam score

| **Interactions** | ***β* ± *SE* in %** | **95% CI** | ***t*-value** | ***p*-value** | **Effect size (*η_p_^2^*)** |
| --- | --- | --- | --- | --- | --- |
| group x TAI-G lack of trust | 0.61 ± 0.67 | -0.71, 1.93 | 0.90 | .371 | 0.008 |
| group x TAI-G interference | 0.72 ± 0.53 | -0.32, 1.76 | 1.36 | .176 | 0.018 |

Note. Degrees of freedom (df) = 103. *β*s represent estimated mean differences. *SE*, standard error; CI, confidence interval; *η_p_^2^*, partial eta squared, TAI-G, German Test Anxiety Inventory to assess test anxiety.

**Supplementary Table S4** Interaction effects between group and resource-related learning strategies on exam score

| **Interactions** | ***β* ± *SE* in %** | **95% CI** | ***t*-value** | ***p*-value** | **Effect size (*η_p_^2^*)** |
| --- | --- | --- | --- | --- | --- |
| group x resource-related LS attention | 3.23 ± 2.89 | -2.45, 8.90 | 1.11 | .268 | 0.012 |
| group x resource-related LS learning context | 2.57 ± 2.27 | -1.88, 7.03 | 1.13 | .261 | 0.013 |
| group x resource-related LS peer learning | 1.82 ± 2.02 | -2.14, 5.78 | 0.90 | .369 | 0.008 |
| group x resource-related LS literature | 2.10 ± 1.95 | -1.73, 5.93 | 1.07 | .285 | 0.012 |

Note. Degrees of freedom (df) = 103. *β*s represent estimated mean differences. *SE*, standard error; CI, confidence interval; *η_p_^2^*, partial eta squared; LS, Learning Strategies, assessed by means of LIST (Inventory for Learning Strategies; German: Inventar zur Erfassung von Lernstrategien im Studium).

**Supplementary Table S5** Interaction effects between group, test anxiety and learning strategies on exam score

| **Interactions** | ***β* ± SE in %** | **95% CI** | ***t*-value** | ***p*-value** | **Effect size (*η_p_^2^*)** |
| --- | --- | --- | --- | --- | --- |
| group x TAI-G x cognitive LS | -0.06 ± 0.07 | -0.19, 0.08 | -0.80 | .425 | 0.007 |
| group x TAI-G x metacognitive LS | -0.16 ± 0.25 | -0.66, 0.34 | -0.64 | .525 | 0.004 |
| group x TAI-G x resource-related LS | -0.03 ± 0.06 | -0.15, 0.09 | -0.50 | .616 | 0.003 |

Note. Degrees of freedom (df) = 103. *β*s represent estimated mean differences; *SE*, standard error; CI, confidence interval; *η_p_^2^* = partial eta squared; TAI-G = German Test Anxiety Inventory; LS = Learning Strategies, assessed by means of LIST (Inventory for Learning Strategies; German: Inventar zur Erfassung von Lernstrategien im Studium).

**Supplementary Table S6** Interaction effects between group, test anxiety and additional exploratory covariates on exam scores

| **Interactions** | ***β* ± *SE* in %** | **95% CI** | ***t*-value** | ***p*-value** | **Effect size (*η_p_^2^*)** |
| --- | --- | --- | --- | --- | --- |
| group x TAI-G x biological sex | -0.12 ± 0.32 | -0.74, 0.51 | -0.37 | .713 | 0.001 |
| group x TAI-G x PDQ20 | < 0.01 ± 0.01 | -0.01, 0.02 | 0.18 | .859 | < 0.001 |
| group x TAI-G x SOMS-7 symptom count | < 0.01 ± 0.02 | -0.04, 0.05 | 0.06 | .949 | < 0.001 |
| group x TAI-G x SOMS-7 symptom intensity | < 0.01 ± 0.01 | -0.02, 0.024 | 0.17 | .866 | < 0.001 |
| group x TAI-G x STAI-S | -0.01 ± 0.02 | -0.04, 0.02 | -0.60 | .551 | 0.004 |
| group x TAI-G x POMS | < 0.01 ± 0.005 | -0.010, 0.011 | 0.12 | .907 | < 0.001 |
| group x TAI-G x neuroticism | 0.26 ± 0.45 | -0.62, 1.13 | 0.58 | .566 | 0.003 |
| group x TAI-G x extraversion | 0.09 ± 0.60 | -1.08, 1.27 | 0.16 | .874 | < 0.001 |
| group x TAI-G x openness | 0.20 ± 0.62 | -1.02, 1.43 | 0.32 | .746 | 0.001 |
| group x TAI-G x conscientiousness | 0.48 ± 0.62 | -0.74, 1.71 | 0.77 | .442 | 0.006 |
| group x TAI-G x agreeableness | -0.23 ± 0.48 | -1.18, 0.71 | -0.48 | .628 | 0.002 |
| group x TAI-G x IE4 internal locus | 0.30 ± 0.30 | -0.28, 0.88 | 1.02 | .310 | 0.011 |
| group x TAI-G x IE4 external locus | 0.05 ± 0.20 | -0.34, 0.45 | 0.26 | .799 | < 0.001 |
| group x TAI-G x CEQ credibility | 0.04 ± 0.05 | -0.06, 0.14 | 0.71 | .478 | 0.005 |
| group x TAI-G x CEQ expectancy | 0.07 ± 0.07 | -0.06, 0.20 | 1.07 | .286 | 0.012 |

Note. Degrees of freedom (df) = 103. *β*s represent estimated mean differences; *SE*, standard error; CI, confidence interval; *η_p_^2^* = partial eta squared; TAI-G = German Test Anxiety Inventory; PDQ20 = Perceived Stress Questionnaire; SOMS-7 = Screening of Somatoform Disorders-7; STAI-S = State and Trait Anxiety Inventory-Stated; POMS = Profile of Mood State; IE4 = Internal–External Locus of Control Short Scale–4; CEQ = Credibility and Expectancy Questionnaire.

**Supplementary Table S7** Interaction effects between group, learning strategies and additional exploratory covariates on exam score

| **Interactions** | ***β* ± *SE* in %** | **95% CI** | ***t*-value** | ***p*-value** | **Effect size (*η_p_^2^*)** |
| --- | --- | --- | --- | --- | --- |
| group x cognitive LS x biological sex | 0.12 ± 1.49 | -2.79, 3.04 | 0.08 | .934 | < 0.001 |
| group x cognitive LS x PDQ20 | -0.02 ± 0.05 | -0.12, 0.08 | -0.41 | .681 | 0.002 |
| group x cognitive LS x STAI-S | -0.002 ± 0.11 | -0.22, 0.21 | - 0.01 | .988 | < 0.001 |
| group x cognitive LS x POMS | -0.04 ± 0.05 | -0.14, 0.06 | - 0.83 | .411 | 0.007 |
| group x cognitive LS x neuroticism | 0.61 ± 3.00 | -5.26, 6.48 | 0.20 | .839 | < 0.001 |
| group x cognitive LS x extraversion | 0.92 ± 2.96 | -4.88, 6.72 | 0.31 | .756 | 0.001 |
| group x cognitive LS x openness | 0.08 ± 2.44 | -4.70, 4.86 | 0.03 | .974 | < 0.001 |
| group x cognitive LS x conscientiousness | -2.40 ± 3.60 | -9.46, 4.66 | -0.67 | .506 | 0.005 |
| group x cognitive LS x agreeableness | 2.57 ± 2.42 | -2.18, 7.32 | 1.06 | .291 | 0.011 |
| group x cognitive LS x IE4 internal locus | -0.18 ± 1.53 | -3.20, 2.82 | -0.12 | .906 | < 0.001 |
| group x cognitive LS x IE4 external locus | 1.27 ± 1.04 | -0.76, 3.31 | 1.23 | .222 | 0.015 |
| group x cognitive LS x CEQ credibility | -0.46 ± 0.32 | -1.10, 0.17 | -1.42 | .158 | 0.021 |
| group x cognitive LS x CEQ expectancy | -0.52 ± 0.32 | -1.14, 0.10 | -1.65 | .102 | 0.027 |
| group x metacog LS x biological sex | -5.04 ± 5.15 | -15.14, 5.05 | -0.98 | .330 | 0.010 |
| group x metacog LS x PDQ20 | 0.19 ± 0.20 | -0.19, 0.57 | 0.97 | .336 | 0.009 |
| group x metacog LS x SOMS-7 sympt. count | 0.86 ± 0.70 | -0.52, 2.24 | 1.23 | .223 | 0.016 |
| group x metacog LS x SOMS-7 sympt. intensity | 0.35 ± 0.49 | -0.62, 1.31 | 0.70 | .483 | 0.005 |
| group x metacog LS x STAI-S | 0.52 ± 0.39 | -0.24, 1.28 | 1.35 | .181 | 0.019 |
| group x metacog LS x POMS | -0.04 ± 0.19 | -0.42, 0.33 | -0.22 | .825 | < 0.001 |
| group x metacog LS x neuroticism | 8.47 ± 9.94 | -11.01, 27.9 | 0.85 | .396 | 0.008 |
| group x metacog LS x extraversion | 3.21 ± 9.32 | -15.06, 21.5 | 0.35 | .731 | 0.001 |
| group x metacog LS x openness | -0.82 ± 9.13 | -18.7, 17.08 | -0.09 | .929 | < 0.001 |
| group x metacog LS x conscientiousness | -4.52 ± 9.93 | -24.9, 14.9 | -0.46 | .650 | 0.002 |
| group x metacog LS x agreeableness | 4.18 ± 8.75 | -13.0, 21.3 | 0.48 | .634 | 0.002 |
| group x metacog LS x IE4 internal locus | -0.46 ± 5.93 | -12.08, 11.15 | -0.08 | .938 | < 0.001 |
| group x metacog LS x IE4 external locus | 4.09 ± 4.23 | -4.2, 12.4 | 0.97 | .336 | 0.009 |
| group x metacog LS x CEQ credibility | -0.32 ± 1.10 | -2.48, 1.84 | -0.29 | .771 | < 0.001 |
| group x metacog LS x CEQ expectancy | -0.39 ± 1.28 | -2.9, 2.12 | -0.30 | .762 | < 0.001 |
| group x resource LS x biological sex | -0.24 ± 1.38 | -2.94, 2.46 | -0.17 | .862 | < 0.001 |
| group x resource LS x PDQ20 | 0.04 ± 0.04 | -0.05, 0.13 | 0.82 | .412 | 0.007 |
| group x resource LS x SOMS-7 sympt. count | 0.08 ± 0.15 | -0.20, 0.37 | 0.57 | .567 | 0.003 |
| group x resource LS x SOMS-7 sympt. intensity | 0.05 ± 0.08 | -0.11, 0.22 | 0.60 | .548 | 0.004 |
| group x resource LS x STAI-S | 0.02 ± 0.09 | -0.15, 0.20 | 0.27 | .788 | < 0.001 |
| group x resource LS x POMS | <0.01 ± 0.04 | -0.07, 0.07 | 0.08 | .933 | < 0.001 |
| group x resource LS x neuroticism | 0.13 ± 2.59 | -4.95, 5.20 | 0.05 | .961 | < 0.001 |
| group x resource LS x extraversion | -1.10 ± 2.48 | -5.96, 3.76 | -0.44 | .659 | 0.002 |
| group x resource LS x openness | 0.57 ± 2.26 | -3.86, 5.0 | 0.25 | .801 | < 0.001 |
| group x resource LS x conscientiousness | -3.11 ± 2.52 | -8.05, 1.8 | -1.23 | .220 | 0.016 |
| group x resource LS x agreeableness | 2.88 ± 2.11 | -1.25, 7.02 | 1.37 | .175 | 0.019 |
| group x resource LS x IE4 internal locus | 0.19 ± 1.33 | -2.4, 2.8 | 0.14 | .887 | < 0.001 |
| group x resource LS x IE4 external locus | 0.52 ± 0.96 | -1.37, 2.4 | 0.54 | .589 | 0.003 |
| group x resource LS x CEQ credibility | -0.09 ± 0.24 | -0.56, 0.39 | -0.36 | .722 | 0.001 |
| group x resource LS x CEQ expectancy | 0.07 ± 0.28 | -0.48, 0.61 | 0.24 | .813 | < 0.001 |
| group x Cognitive organization x SOMS-7 count | 0.66 ± 0.42 | -0.17, 1.48 | 1.56 | .122 | 0.025 |
| group x Cognitive organization x SOMS-7 intensity | 0.29 ± 0.30 | -0.3, 0.87 | 0.97 | .336 | 0.009 |
| group x Cognitive elaboration x SOMS-7 count | 0.72 ± 0.60 | -0.46, 1.90 | 1.19 | .237 | 0.015 |
| group x Cognitive elaboration x SOMS-7 intensity | 0.41 ± 0.35 | -0.27, 1.09 | 1.18 | .243 | 0.014 |
| group x Cognitive critical thinking x SOMS-7 count | 0.56 ± 0.40 | -0.23, 1.35 | 1.38 | .171 | 0.019 |
| group x Cognitive critical thinking x SOMS-7 intensity | 0.27 ± 0.22 | -0.17, 0.69 | 1.18 | .242 | 0.014 |
| group x Cognitive rehearsal x SOMS-7 count | 0.27 ± 0.46 | -0.6, 1.17 | 0.59 | .559 | 0.003 |
| group x Cognitive rehearsal x SOMS-7 intensity | 0.15 ± 0.30 | -0.43, 0.73 | 0.52 | .604 | 0.003 |

Note. Degrees of freedom (df) = 103. *β*s represent estimated mean differences; *SE*, standard error; CI, confidence interval; *η_p_^2^*, partial eta squared; LS, Learning Strategies, assessed by means of LIST (Inventory for Learning Strategies; German: Inventar zur Erfassung von Lernstrategien im Studium); PDQ20, Perceived Stress Questionnaire; STAI-S, State and Trait Anxiety Inventory, State; POMS, Profile of Mood State; IE4, Internal–External Locus of Control Short Scale–4; CEQ, Credibility and Expectancy Questionnaire; SOMS-7, Screening of Somatoform Disorders-7.

**Supplementary Figure S1**

**

**

Figure S1. Correlation between cognitive learning strategies (LS) and exam scores for the control group (CTR, left) and the open label placebo (OLP) group (right), respectively. Blue regression lines represent correlations at different levels of somatic symptoms (i.e., mean ± 1standard deviation (SD); A: somatic symptoms count, B: intensity of somatic symptoms), including individual data points and 95% confidence intervals (shaded bands).

**References**

1. Hodapp, V. Das Prüfungsängstlichkeitsinventar TAI-G: Eine erweiterte und modifizierte Version mit vier Komponenten. *Zeitschrift für Pädagogische Psychologie/German Journal of Educational Psychology* (1991).
2. Harpell, J. V. & Andrews, J. J. W. Multi-Informant Test Anxiety Assessment of Adolescents. *PSYCH* **03**, 518–524 (2012).
3. Wild, K.-P., & Schiefele, U. (1994). Lernstrategien im Studium: ergebnisse zur Faktorenstruktur und Reliabilität eines neuen Fragebogens. *Zeitschrift für differentielle und diagnostische Psychologie*.
4. Wild K.P., Schiefele U., Winteler A. *LIST. Ein Verfahren Zur Erfassung von Lernstrategien Im Studium.* (Universität der Bundeswehr, Institut für Erziehungswissenschaft und Pädagogische Psychologie, 1992).
5. Duncan, T. G. & McKeachie, W. J. The Making of the Motivated Strategies for Learning Questionnaire. *Educational Psychologist* **40**, 117–128 (2005).
